# Efficacy and safety of intermittent vs continuous furosemide for heart failure concomitant renal dysfunction: A protocol for systematic review and meta-analysis

**DOI:** 10.1097/MD.0000000000025669

**Published:** 2021-04-23

**Authors:** Siqi Chen, Yan Lin, Meiyang Zhou, Canxin Zhou

**Affiliations:** Department of Nephrology, The Affiliated People's Hospital, Ningbo University, Ningbo, China.

**Keywords:** continuous furosemide, heart failure, intermittent furosemide, meta-analysis, protocol, renal dysfunction

## Abstract

**Background:**

Currently, there are no meta-analyses evaluating the efficacy and safety of intermittent vs continuous furosemide for heart failure concomitant renal dysfunction. Our protocol is conceived to evaluate the efficacy and safety of intermittent vs continuous furosemide for heart failure concomitant renal dysfunction.

**Methods:**

We will follow the Preferred Reporting Items for Systematic Reviews and Meta-Analyses (PRISMA) reporting guidelines and the recommendations of the Cochrane Collaboration to conduct this meta-analysis. The systematic review protocol has been registered in Open Science Framework registries. The following databases including PubMed, Cochrane Library, Web of Science, and EMBASE will be searched using the key phrases “loop diuretics,” “furosemide,” “heart failure,” and “renal dysfunction” for all randomized clinical trials (RCTs) published up to May 2021. Revman 5.3 (Nordic Cochrane Centre, Denmark) will be used to complete the meta-analysis and generate forest plots. We will choose between a fixed effects and random effects model based upon the heterogeneity of included studies. Significance will be set at *P* < .05.

**Results:**

Our protocol is conceived to test the hypothesis that continuous furosemide could lead to better outcomes in patients presenting with heart failure concomitant renal dysfunction.

**Registration number:**

10.17605/OSF.IO/CQZRS.

## 1 Introduction

Renal dysfunction is closely related to the aggravation of heart failure. It is also associated with poor long-term outcomes in heart failure patients with chronic kidney disease, according to a study from the Japanese Cardiac Registry of Heart Failure in Cardiology.^[1]^ Previous studies have shown that deteriorating kidney function, defined as an increase in serum creatinine levels in the hospital, may be a strong predictor of increased mortality.^[2,3]^ Additionally, previous trials have shown that medium-term deterioration of renal function (defined as changes in serum creatinine within 6 months) predicts an increase in mortality.^[4,5]^

Loop diuretics are widely used and are essential in the treatment of heart failure patients with symptoms of fluid overload. However, previous studies have reported that a higher dose of loop diuretics is an independent predictor of worsening renal function, and that loop diuretic use is dose-dependent with mortality.^[6,7]^ Therefore, the deterioration of cardiorenal syndrome due to high dose of loop diuretics is of concern. To maintain high concentrations of furosemide delivery in the proximal renal tubule to allow it to effectively act as a Na-K-2CL transporter on the luminal side of the thick ascending limb of the loop of Henle, optimizing the dose of furosemide is required when glomerular filtration rate is compromised.^[8,9]^

Several clinical trials have concluded that continuous loop diuretics produce a better diuretic effect than intermittent loop diuretics at the same total dose.^[10–12]^ Possible explanations include constant urine output and less neurohormonal activation due to the continuous delivery rate of furosemide to the tubules and lower drug peaks, which result in less intravascular volume changes and fewer serious adverse events. Currently, there are no meta-analyses evaluating the efficacy and safety of intermittent vs continuous furosemide for heart failure concomitant renal dysfunction. Our protocol is conceived to evaluate the efficacy and safety of intermittent vs continuous furosemide for heart failure concomitant renal dysfunction and test the hypothesis that continuous furosemide could lead to better outcomes in patients presenting with heart failure concomitant renal dysfunction.

## 2 Materials and methods

### 2.1 Search strategy

We will follow the Preferred Reporting Items for Systematic Reviews and Meta-Analyses (PRISMA) reporting guidelines and the recommendations of the Cochrane Collaboration to conduct this meta-analysis. The systematic review protocol has been registered in Open Science Framework registries. The following databases including PubMed, Cochrane Library, Web of Science, and EMBASE will be searched using the key phrases “loop diuretics,” “furosemide,” “heart failure,” and “renal dysfunction” for all randomized clinical trials (RCTs) published up to May 2021. Ethical approval is not necessary because the present meta-analysis will be performed based on previous published studies.

### 2.2 Eligibility criteria

Study included in our meta-analysis has to meet all of the following inclusion criteria in the PICOS order:

1. Population: patients with heart failure concomitant renal dysfunction;
2. Intervention: patients receive continuous furosemide;
3. Comparison intervention: patients receive intermittent furosemide;
4. Outcome measures: at least one of the following outcome measures was reported: freedom from congestion at 72 hours, net daily urine output, weight loss during the study, total urinary sodium excretion, adverse events, and length of hospital stay;
5. Study design: English language RCT.

Exclusion criteria included the following points:

1. non-English language papers;
2. non-RCTs such as case reports, animal trials, letters, and reviews;
3. conference abstracts and duplicate reports;
4. studies with no data analysis and/or power analysis.

### 2.3 Data extraction

Two independent authors will extract the following descriptive raw information from the selected studies, including inclusion and exclusion criteria, number of patients, detailed intervention protocols, follow-up time, and outcome measures. Disagreements will be resolved through discussion with the third author. The primary outcome is freedom from congestion at 72 hours. Secondary outcome measures include net daily urine output, weight loss during the study, total urinary sodium excretion, adverse events, and length of hospital stay. If the data are missing or can not be extracted directly, we will contact the corresponding authors to ensure that the information is integrated. Otherwise, we will calculate them with the guideline of Cochrane Handbook for Systematic Reviews of Interventions 5.1.0. If necessary, we will abandon the extraction of incomplete data.

### 2.4 Data analysis

Revman 5.3 (Nordic Cochrane Centre, Denmark) will be used to complete the meta-analysis and generate forest plots. We will use the Mantel–Haenzel method to calculate the pooled odds ratio. Odds ratio with a 95% confidence interval and mean difference or standardized mean differences with 95% confidence interval will be assessed for dichotomous outcomes or continuous outcomes, respectively. The heterogeneity will be assessed by using the Q test and *I*^2^ statistic. An *I*^2^ value of <25% is chosen to represent low heterogeneity and an *I*^2^ value of >75% to indicate high heterogeneity. We will choose between a fixed effects and random effects model based upon the heterogeneity of included studies. Significance will be set at *P* < .05.

### 2.5 Assessment of methodological quality

In order to achieve a consistency (at least 80%) of risk of bias assessment, the risk of bias assessors will preassess a sample of eligible studies. Results of the pilot risk of bias will be discussed among review authors and assessors. Two independent reviewers will assess the risk of bias of the included studies at study level. We will follow the guidance in the latest version of Cochrane Handbook for systematic reviews of interventions when choosing and using tools to assessing risk of bias for randomized trials (version 2 of the Cochrane risk of bias tool for randomized trials, RoB 2) and nonrandomized trials (the Risk Of Bias In Nonrandomized Studies of Interventions, ROBINS-I tool). Any disagreements will be discussed and resolved in discussion with a third reviewer. Studies with high risk of bias or unclear bias will be given less weight in our data synthesis.

## 3 Discussion

Loop diuretics, such as furosemide, have long been used to treat fluid overload in patients with congestive heart failure. However, they have numerous adverse effects, including low blood pressure, electrolyte disorders, worsening renal function, and worsening nitrogen metabolism. Several clinical trials have concluded that continuous loop diuretics produce a better diuretic effect than intermittent loop diuretics at the same total dose.^[10–12]^ Possible explanations include constant urine output and less neurohormonal activation due to the continuous delivery rate of furosemide to the tubules and lower drug peaks, which result in less intravascular volume changes and fewer serious adverse events. Currently, there are no meta-analyses evaluating the efficacy and safety of intermittent vs continuous furosemide for heart failure concomitant renal dysfunction. Our protocol is conceived to evaluate the efficacy and safety of intermittent vs continuous furosemide for heart failure concomitant renal dysfunction and test the hypothesis that continuous furosemide could lead to better outcomes in patients presenting with heart failure concomitant renal dysfunction.

## Author contributions

**Conceptualization:** Meiyang Zhou.

**Data curation:** Siqi Chen, Yan Lin.

**Formal analysis:** Siqi Chen, Yan Lin.

**Funding acquisition:** Canxin Zhou.

**Investigation:** Siqi Chen, Yan Lin.

**Methodology:** Yan Lin, Meiyang Zhou.

**Project administration:** Canxin Zhou.

**Resources:** Meiyang Zhou, Canxin Zhou.

**Software:** Siqi Chen, Meiyang Zhou.

**Supervision:** Canxin Zhou.

**Visualization:** Meiyang Zhou.

**Writing – original draft:** Siqi Chen, Yan Lin.

**Writing – review & editing:** Canxin Zhou.
